# Clinicopathological and Prognostic Role of Long Noncoding RNA Linc00152 in Various Human Neoplasms: Evidence from Meta-Analysis

**DOI:** 10.1155/2017/6010721

**Published:** 2017-11-23

**Authors:** Chenkui Miao, Kai Zhao, Jundong Zhu, Chao Liang, Aiming Xu, Yibo Hua, Jianzhong Zhang, Shouyong Liu, Ye Tian, Chao Zhang, Yuhao Wang, Shifeng Su, Zengjun Wang, Bianjiang Liu

**Affiliations:** ^1^State Key Laboratory of Reproductive Medicine and Department of Urology, The First Affiliated Hospital of Nanjing Medical University, Nanjing, China; ^2^Department of Urology, Nanjing First Hospital, Nanjing Medical University, Nanjing, Jiangsu, China

## Abstract

Recent researches have demonstrated that long noncoding RNA linc00152 was aberrantly upregulated in multiple tumor types. High expression of linc00152 was associated with poor outcomes in cancer patients. Therefore, we conducted this meta-analysis to evaluate its potential value as a prognostic predictor in various human neoplasms. Eligible studies were searched through several electronic databases including PubMed, Embase, Web of Science, and the Cochrane Library. Eight original studies including 752 cancer patients were ultimately enrolled. Statistical analysis suggested that overexpression of linc00152 was significantly correlated with unfavorable overall survival (OS) (HR = 2.05, 95% CI: 1.59–2.64) and disease-free/progression-free survival (DFS/PFS) (HR = 3.52, 95% CI: 1.82–6.79) in cancer patients. In addition, a significant correlation was observed between aberrant linc000152 expression and lymph node metastasis (LNM) (OR = 2.49, 95% CI: 1.57–3.94) but not in vessel invasion (VI) (OR = 1.02, 95% CI: 0.54–1.93) and distant metastasis (DM) (OR = 0.600, 95% CI: 0.213–1.689). Our meta-analysis demonstrated that high linc00152 expression significantly predicted inferior OS and DFS/PFS in multiple neoplasms, as well as advanced LNM and VI. Linc00152 may serve as a potential indicator in predicting poor outcomes and metastases of diverse cancers.

## 1. Introduction

With the rapid progress of RNA-sequencing technology, long noncoding RNA has greatly drawn more and more attention of society [1, 2]. LncRNAs were one class of non-protein-coding RNA molecules, which are greater than 200 nucleotides in length, without open reading frame [3–5]. To our knowledge, accumulating studies have detected a variety of aberrant expression lncRNAs in multiple human cancers. In spite the “junk” of genome lncRNAs being considered as insignificant, they are now confirmed to exert pivotal roles in tumor biological processes, including carcinogenic or antitumor effects via transcriptional and posttranscriptional regulations [6, 7]. Furthermore, emerging evidence has indicated that the abnormal expression of lncRNAs was correlated with progression of neoplasms, including clinical-pathological features and prognostic outcomes [7–12]. Therefore, lncRNA can serve as a potential biomarker for predicting progression and survival in patients with carcinomas. To better understand the tumor mechanism at lncRNA level, it is of primary necessity to predict potential disease-lncRNA association, which could also benefit the detection of novel biomarkers for cancer treatment and prognosis. However, in previous years, there have been very limited computational models to experimentally confirm the disease-lncRNA associations.

In recent years, lncRNA linc00152 has caught great attention due to its involvement in multiple cancers. In 2013 Cao et al. generated the lncRNA expression profiles in gastric cancer by using robust multiarray average method, and first reported the differentially expressed lncRNA linc00152 in tumor tissues [13]. It has been showed that linc00152 was overexpressed in multiple types of cancers, including gastric cancer, renal cell carcinoma, gallbladder cancer, lung adenocarcinoma, and hepatocellular carcinoma [14–16]. In addition, knocking down linc00152 expression could inhibit cell migration, invasion, and proliferation and induce cell cycle arrest in G0/G1 and cell apoptosis in vitro [17, 18]. Elevated linc00152 expression was also found to be significantly associated with unfavorable prognosis in cancer patients. For instance, Hu et al. demonstrated that linc00152 expression was upregulated in ESCC specimens and high linc00152 level predicted shorter survival time [19]. Cai et al. have confirmed that linc00152 was correlated with poorer overall survival, as well as advanced lymph node metastasis and vessel invasion in gallbladder cancer patients [20]. However, certain investigations have presented the adverse results. Qiu and Yan declared that high linc00152 expression was related to superior overall survival in patients with colorectal carcinoma by using GSE dataset [21]. The discrepancies between these studies highlight the importance of evaluating the prognostic significance of linc00152 in human malignant neoplasms. Therefore, we carried out this systematic review and meta-analysis to clarify the predictive value of linc00152 in cancer patients.

## 2. Materials and Methods

### 2.1. Search Strategy

The literature retrieval was carried out by two independent reviewers (Chenkui Miao and Kai Zhao) through electronic databases including PubMed, Embase, Web of Science, and the Cochrane Library up to March 2017. The combinations of key words were used as follows: (“long non-coding RNA linc00152” or “lncRNA linc00152” or “linc00152”) and (“cancer” or “carcinoma” or “neoplasm” or “tumor” or “malignancy”). The following criteria were utilized to screen appropriate articles: (1) English publications, (2) studies focusing on multiple malignancies; (3) associations between linc00152 and clinical outcomes. In order to supplement our literature search, the reference lists of relevant articles were observed for additional eligible studies.

### 2.2. Quality Assessment

**Figure 1:**
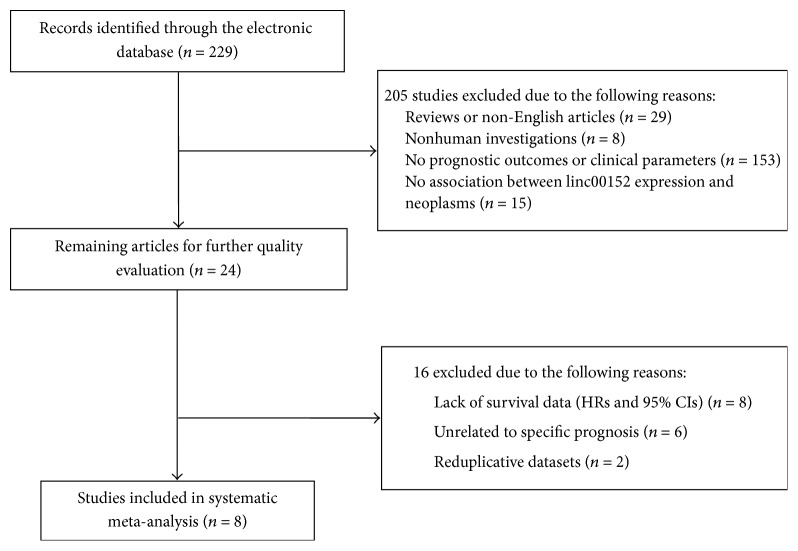
Flow diagram of the study selection process.

We used a critical review checklist to evaluate the quality of all included studies. Quality assessment should contain the following criteria: (1) the study country and population, (2) the study design, (3) the detection of linc00152, (4) the cut-off points of linc00152, (5) the samples and pathology information, and (6) the clinical outcomes and follow-up duration. Articles were excluded when they did not cover the points above. A flow diagram of the study selection process is presented in Figure 1.

### 2.3. Data Extraction

Two investigators independently extracted relevant data from included studies to rule out any discrepancy. Extracted data elements included the following records: (1) the first authors and year of publication, (2) the study nationality, (3) main ethnicity and cancer types, (4) sample and pathology type, (5) the cut-off value and assay method, (6) following-up months, (7) the case number of linc00152 expression, and (8) HRs, 95% CI, and *p* value for prognostic outcomes [22]. Those indirectly reported HRs and 95% CIs were extracted from graphical survival plots using Engauge Digitizer version 4.1 [23, 24].

### 2.4. Statistical Analysis

With the aim of testing the heterogeneity of pooled HRs, Cochran's *Q*-test and Higgins *I*^2^ statistics (*I*^2^) were performed in the meta-analysis. A fixed-effects model (Mantel-Haenszel method) or random-effects model (DerSimonian-Laird method) was conducted in accordance with the heterogeneity of included studies. When *p* < 0.05 or the percentage of I2 was greater than 75%, a random-effects model was used to analyze the combined HR. Otherwise, a fixed-effects model was applied to analyses. Furthermore, in order to reduce the sources of heterogeneity, we also exerted subgroup analysis depending on various elements. Egger et al.'s test was utilized to detect the publication bias [25, 26]. Stata version 12.0 (Stata Corporation, College Station, TX, USA) was used to calculate all statistical analyses.

## 3. Results

### 3.1. Characteristics of Enrolled Studies

A total of 229 studies were initially retrieved from online database including PubMed, Embase, Web of science, and the Cochrane Library. After a manual screening of titles and abstracts, 205 studies were excluded according to the following reasons: reviews or non-English articles, nonhuman investigations, no association between linc00152 expression and human neoplasms and no prognostic outcomes or clinical parameters. After further screening of the remaining 24 studies, 16 were excluded due to lack of survival data, being unrelated to specific prognosis, or being reduplicative datasets. Finally, eight available studies were deemed applicable to the meta-analysis. The inclusion and exclusion reasons of candidate studies are presented in detail in Figure 1.

Tables 1 and 2 summarize the main characteristics of the selected 8 studies published from 2015 to 2017. The patients number of eight studies ranged from 35 to 205, with a mean subject size of 94. Among the eight studies, six studies reported patients' OS; one focused on DFS and PFS, respectively. Five studies estimated the relationship between linc00152 and LNM, as well as four focusing on VI. The malignant neoplasms investigated consisted of gallbladder cancer, lung adenocarcinoma, and clear cell renal cell carcinoma (ccRCC), esophageal squamous cell carcinoma (ESCC), hepatocellular carcinoma (HCC), colon cancer, and gastric cancer. All included studies were conducted in China and focused on Asian population. Tissue specimens were used to determine linc00152 expression in all studies except one in plasma samples. All of these studies were retrospective in design. Quantitative real-time PCR (qRT-PCR) was widely applied to determine the linc00152 expression.

### 3.2. Association between Linc00152 Expression and OS

**Figure 2:**
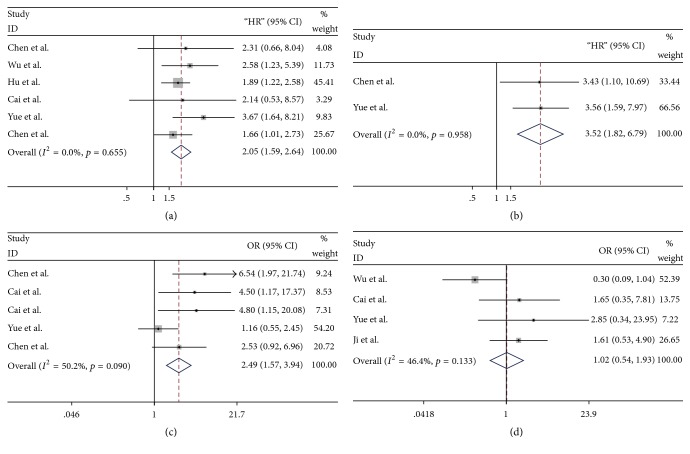
Forest plots of combined analyses associated with linc00152 expression. (a) Overall survival (OS); (b) disease-free survival/progression-free survival (DFS/PFS); (c) lymph node metastasis (LNM); (d) vessel invasion (VI).

**Figure 3:**
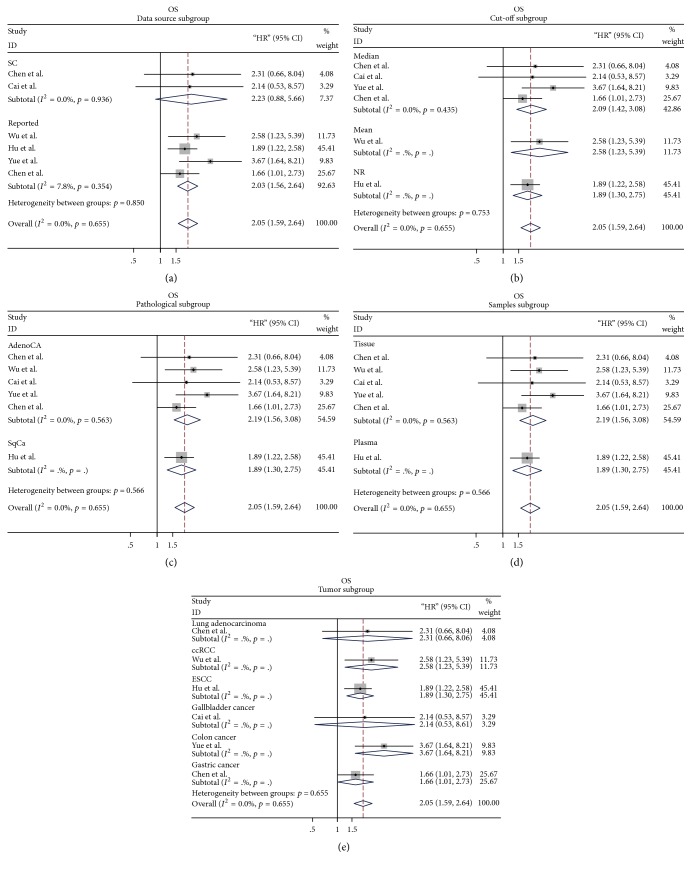
Forest plots of stratified analysis of the OS (a) stratified by data source subgroup; (b) stratified by cut-off subgroup; (c) stratified by pathological subgroup; (d) stratified by sample subgroup; (e) stratified by tumor subgroup.

A total of six original studies were performed to analyze the OS, with a fixed-effects model on account of no obvious heterogeneity (*p* = 0.655, *I*^2^ = 0%). Our meta-analysis indicated that high expression of linc00152 was correlated with poorer OS in patients with multiple carcinomas (HR = 2.05, 95% CI: 1.59–2.64; Figure 2(a)). In stratified analysis, we found that analyses for data from articles reported obtained a significant result between linc00152 and patients survival (HR = 2.03, 95% CI: 1.56–2.64), while those extracted from survival plots failed to get a meaningful outcome (HR = 2.33, 95% CI: 0.88–5.66; Figure 3(a)). Stratification analyses for other subgroups were presented in detail in Figures 3(b)–3(e).

### 3.3. Association between Linc00152 Expression and DFS/PFS

In total, there were 2 studies including 196 patients investigating the prognostic significance of linc00152 on cancer progression or recurrence, with a pooled HR of 3.52 (95% CI: 1.82–6.79; Figure 2(b)). This result demonstrated that linc00152 overexpression predicted higher risk of cancer progression. Given no heterogeneity among the two studies, a fixed-effects model was performed to the calculation (*p* = 0.958, *I*^2^ = 0%). Among them, one focused on the prognostic value of linc00152 expression on cancer relapse (HR = 3.56, 95% CI: 1.59–7.97), and another one explored the association between linc00152 and tumor progression (HR = 3.43, 95% CI: 1.10–10.69). Linc00152 might serve as a crucial indicator for cancer progression.

### 3.4. Association between Linc00152 Expression and Clinicopathological Parameters

A total of five studies comprising 369 cancer patients reported the correlation of linc00152 with LNM in multiple neoplasms. Since no significant heterogeneity was observed among these studies (*p* = 0.090, *I*^2^ = 50.2%), a fixed-effect model was utilized. The combined OR with 95% CI suggested that tumor tissues with high linc00152 expression of cancer patients preferentially metastasize to the lymph nodes (OR = 2.49, 95% CI: 1.57–3.94; Figure 2(c)).

Furthermore, four studies with 355 patients declared the relationship between linc00152 expression levels and vessel invasion of cancer patients, with a fixed-effects model due to no obvious heterogeneity (*p* = 0.133, *I*^2^ = 46.4%). Our results failed to demonstrate any significant association between aberrant linc00152 level and vessel invasion tendency (OR = 1.02, 95% CI: 0.54–1.93; Figure 2(d)). Only one study conducted the DM analysis of cancer patients, indicating that high linc00152 level could not accurately predict the occurrence of DM (OR = 0.600, 95% CI: 0.213–1.689; data not shown). This might be attributed to the limitation of large-scale investigations.

### 3.5. Publication Bias

**Figure 4:**
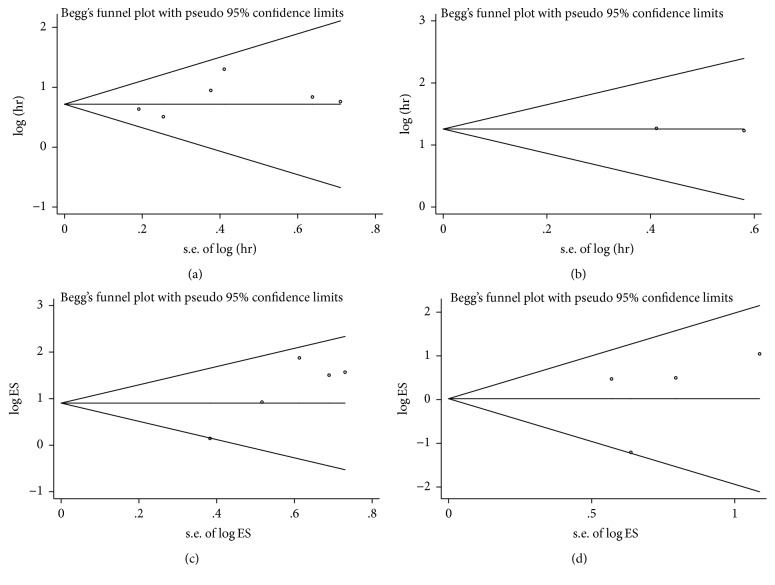
Begg's funnel plots of publication bias test. (a) Overall survival (OS); (b) disease-free survival/progression-free survival (DFS/PFS); (c) lymph node metastasis (LNM); (d) vessel invasion (VI).

Begg's test and Egger's test were used to evaluate the publication bias in this meta-analysis. As is determined in the pooled analyses, Begg's funnel plots with pseudo 95% CIs were symmetric (Figures 4(a)–4(d)). *p* values calculated from Egger's test with higher detection effectiveness were 0.258 for OS, 0.889 for LNM, and 0.612 for VI, respectively, indicating no significant publication bias in the meta-analysis.

### 3.6. Sensitivity Analyses

**Figure 5:**
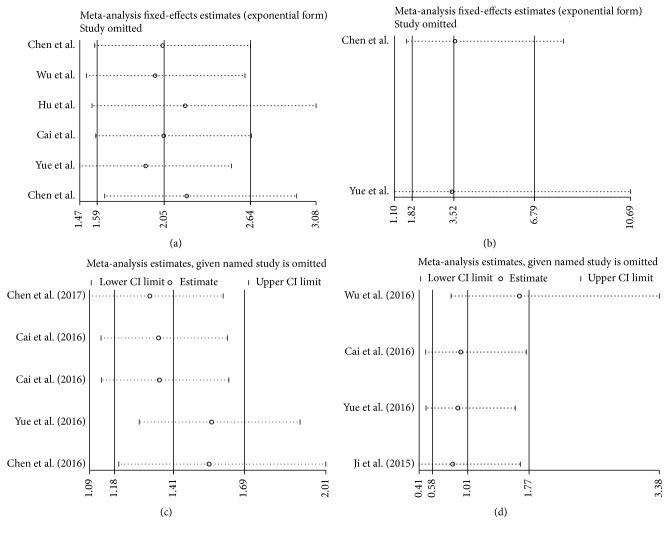
Sensitivity analysis under specific model. (a) Effect of individual studies on the combined HR for OS; (b) effect of individual studies on the combined HR for DFS/PFS; (c) effect of individual studies on the pooled HR for LNM; (d) effect of individual studies on the pooled HR for VI.

Sensitivity analysis was conducted by Stata12.0 software to assess whether exclusion of any individual study affected the overall results. The analyzed result from a fixed model suggested that our results are comparatively credible and stable (Figures 5(a)–5(d)).

## 4. Discussion

At the present time, significant achievements have been made in detecting promising biomarkers for multiple human carcinomas. Nevertheless, the survival outcome is still worse and has brought a great challenge to the public society. Numerous investigations have disclosed the aberrant expression of lncRNAs in tumor tissues compared with normal specimens [27]. LncRNAs have been reported to be involved in various biological activities such as cell proliferation, cycle, invasion, and metastasis [28–30]. As a kind of noncoding RNA with more than 200 nucleotides in length, lncRNAs lack the capacity of protein-coding. Recently, numerous studies have proposed its promising value as biomarkers for early tumor screening and prognosis, as well as clinical therapy for various types of carcinomas [31–33].

Long noncoding linc00152, one of the lncRNAs members, has been shown to be abnormally expressed in diverse types of cancer, such as gastric cancer, renal cell carcinoma, and gallbladder cancer, lung adenocarcinoma, and hepatocellular carcinoma, suggesting that linc00152 may develop a key role in carcinogenesis [14, 15, 17, 18, 20]. Chen et al. detected the linc00152 expression in human lung carcinoma tissues and paired normal tissues by using qRT-PCR and found that linc00152 was upregulated in tumor tissues. Inhibition of linc00152 suppressed cell growth, invasion, and migration and induced cell apoptosis [18]. High expression of linc0152 also predicted advanced TNM stage, larger tumor size, and lymph node metastasis, as well as shorter survival period. Yue et al. found that linc00152 overexpression was associated with poor overall survival and high recurrence risk in colon cancer. Further mechanism investigation revealed that the promoting role of linc00152 mainly depended on miR-193a-3p/ERBB4/AKT signaling axis, which may provide a novel choice confronting the drug resistance [34]. Additionally, in a gallbladder cancer study, evaluated linc00152 expression was found to be positively associated with advanced lymph node metastasis and vessel invasion and negatively correlated with patient's outcomes [20, 35]. However, there were available studies presenting the contradictory summary. By analyzing GSE datasets, Qiu and Yan confirmed that aberrant linc00152 level was an independent factor for unfavorable overall survival [21]. The observation of these antitumor values of linc00152 in cancers might cause doubt on their usual oncogenic function.

In addition, the prognostic and clinicopathological significance of linc00152 might partly be ascribed to its biological activities and molecular mechanisms. Knocking down linc00152 suppressed cell invasion and affects prognosis via interacting with EZH2 and repressing IL24 expression in lung carcinoma [18]. Linc00152 was negatively regulated by miR-376c-3p, restricting cell viability, and stimulates cell apoptosis in colorectal cancer [36]. In addition, Zhao et al. verified linc00152's promoting role in cell cycle arrest, migration, invasion, and epithelial-mesenchymal transition in gastric cancer, through affecting several molecular markers such as N-cadherin, E-cadherin, Vimentin, Slug, and Snail [37]. Considering its complex mechanisms, we speculated that linc00152 might be involved in various biological activities in diverse tumor subtypes. In this meta-analysis, we first assessed the correlation of linc00152 with prognostic outcomes (OS, DFS, and PFS) and clinical parameters (LNM, VI). We also performed subgroup, sensitivity, and heterogeneity analyses to explore the effects of dominant characteristics from available studies. We found that upregulated linc00152 expression induced unfavorable overall survival with a combined HR of 2.05 (95% CI: 1.59–2.64). Stratified analysis of OS demonstrated that data from article reported obtained a significant conclusion when evaluating the role of linc00152 in cancer patients (HR = 2.03, 95% CI: 1.56–2.64). Nevertheless, results for data extracted from survival curves made no sense (HR = 2.33, 95% CI: 0.88–5.66). These discrepancies might be attributed to the inaccuracy of the data from survival plots. In addition, the pooled outcome in the DFS/PFS analysis suggested that increased linc00152 level predicted advanced cancer progression (HR = 3.52, 95% CI: 1.82–6.79). On account of only two studies evaluating the cancer progression with aberrant linc00152 expression in two different types of tumors, it is of no great worth to perform the stratified analyses. Also, what cannot be ignored is the difference of samples between included studies from different lab and institution. This might minimize the reliability of our results at some level. Although samples including in the meta-analysis were differential between studies, a significant association between linc00152 and patients' OS and DFS/PFS could still be reached. However, more high quality and available investigations are needed for further evidence.

Admittedly, numerous studies have shown that linc00152 could promote tumor metastasis and invasion in cancer patients. In the meta-analysis, we demonstrated that high expression of linc00152 was positively correlated with advanced LNM (OR = 2.49, 95% CI: 1.57–3.94); nevertheless abnormal miR-152 expression exerted no statistical significance in VI (OR = 1.02, 95% CI: 0.54–1.93). The deficiencies of studies focusing on linc00152 and clinical characteristics might account for this discrepancy to some extent. Furthermore, the differences between malignancies types also had considerable impacts on its prognostic and clinical role. In this meta-analysis, no significant heterogeneity was observed when we carried out OS and DFS/PFS analysis of linc00152, as well as comparison for LNM and VI. Furthermore, sensitivity analysis was also conducted to enhance the conclusion of this meta-analysis. Exclusion of any individual studies alters little change of the pooled significance. No obvious publication bias was detected in this meta-analysis, indicating our analysis was credible.

Despite the meta-analysis being performed with rigorous statistics, our conclusion still has several limitations for the following reasons. First, all investigations included were published in English, inducing English language bias in combined results [38, 39]. Second, the quantity of included studies was not sufficient for a more comprehensive result. Third, only Asian population were applicable in this meta-analysis, which might minimize the analyzing value to some level. Moreover, difference in samples from different institutions might reduce the credibility of our conclusion, which serves as an unavoidable factor in the study. Regarding these deficiencies, the role of linc00152 in multiple human malignancies might be exceedingly evaluated. Therefore, further large-scale and well-designed studies are needed to verify the function of linc00152 in various carcinomas.

To summarize, this meta-analysis reveals that long noncoding RNA linc00152 can predict poor prognostic and metastasis in multiple neoplasms, especially promoting advanced LNM. Considering the insufficient evidence, on purpose to better evaluate the prognostic role of linc00152 expression in malignant patients, more higher quality researches are required for further confirmation.

## Figures and Tables

**Table 1:** Main characteristics of included studies in the meta-analysis.

Study	Year	Tumor type	Case nationality	Median or mean age	Dominant ethnicity	Study design	Main pathological type	Tumor stage (I/II/III/IV)	Detected sample	Outcome measures	Source of HR	Maximum months of follow-up
Chen et al.	2017	Lung adenocarcinoma	China	65	Asian	R	AdenoCA	24/36 (I-II/IIIa)	Tissue	OS/PFS	SC	40
Wu et al.	2016	ccRCC	China	60	Asian	R	AdenoCA	22/55 (I/II–IV)	Tissue	OS	Reported	80
Hu et al.	2016	ESCC	China	54.29	Asian	R	SqCa	133/72 (I-II/III-IV)	Plasma	OS	Reported	60
Cai et al.	2016	Gallbladder cancer	China	60	Asian	R	AdenoCA	17/23 (I-II/III-IV)	Tissue	NR	NR	NR
Cai et al.	2016	Gallbladder cancer	China	60	Asian	R	AdenoCA	13/22 (I-II/III-IV)	Tissue	OS	SC	40
Yue et al.	2016	Colon cancer	China	65	Asian	R	AdenoCA	66/68 (II/III)	Tissue	OS/DFS	Reported	100
Chen et al.	2016	Gastric cancer	China	60	Asian	R	AdenoCA	32/16 (I-II/III-IV)	Tissue	OS	Reported	60
Ji et al.	2015	HCC	China	60	Asian	R	AdenoCA	61/41 (I-II/III-IV)	Tissue	NR	NR	NR

Study design is described as retrospective (R). AdenoCA, adenocarcinoma; SqCa, squamous carcinoma; OS, overall survival; DFS, disease-free survival; PFS, progression-free survival. ccRCC, clear cell renal cell carcinoma; ESCC, esophageal squamous cell carcinoma; HCC, hepatocellular carcinoma. NR, not reported; SC, survival curve.

**Table 2:** HRs and 95% CIs of cancer prognosis and progression associated with linc00152 expression in included studies.

Study	Year	Tumor type	Main assay method	Cut-off value	Case number of linc00152 expression								OS	*P* value	DFS/PFS	*P* value
					High	High with LNM	High with DM	High with VI	Low	Low with LNM	Low with DM	Low with VI	HR (95% CI) (U/M)		HR (95% CI) (U/M)	
Chen et al.	2017	Lung adenocarcinoma	qRT-PCR	Median	30	25	NR	NR	30	13	NR	NR	2.31 (0.66–8.04) U^*∗*^	0.005	3.43 (1.10–10.69) U^*∗*^	<0.001
Wu et al.	2016	ccRCC	qRT-PCR	Mean	38	NR	8	4	39	NR	12	11	2.577 (1.233–5.387) M	0.012	NR	NR
Hu et al.	2016	ESCC	qRT-PCR	NR	131	NR	NR	NR	74	NR	NR	NR	1.888 (1.220–2.581) M	0.0028	NR	NR
Cai et al.	2016	Gallbladder cancer	qRT-PCR	Median	23	15	NR	6	17	5	NR	3	NR	NR	NR	NR
Cai et al.	2016	Gallbladder cancer	qRT-PCR	Median	18	12	NR	NR	17	5	NR	NR	2.14 (0.53–8.57) U^*∗*^	<0.05	NR	NR
Yue et al.	2016	Colon cancer	qRT-PCR	Median	98	50	NR	7	38	18	NR	1	3.67 (1.64–8.21) M	0.002	3.56 (1.59–7.97) M	0.002
Chen et al.	2016	Gastric cancer	qRT-PCR	Median	49	43	NR	NR	48	34	NR	NR	1.659 (1.008–2.731) M	0.047	NR	NR
Ji et al.	2015	HCC	qRT-PCR	Median	51	NR	NR	9	51	NR	NR	6	NR	NR	NR	NR

The source of HR and 95% CI was extracted from survival curves or article reports. ^*∗*^HR calculated from survival curves; SC, survival curve; U, univariate analysis; M, multivariate analysis; NR, not reported. ccRCC, clear cell renal cell carcinoma; ESCC, esophageal squamous cell carcinoma; HCC, hepatocellular carcinoma. LNM, lymph node metastasis; DM, distant metastasis; VI, vessel invasion; OS, overall survival; DFS, disease-free survival; PFS, progression-free survival. qRT-qPCR, reverse transcriptase-quantitative PCR.
